# Correction to: Predictive landscapes hidden beneath biological cellular automata

**DOI:** 10.1007/s10867-021-09597-2

**Published:** 2021-12-23

**Authors:** Lars Koopmans, Hyun Youk

**Affiliations:** 1 Program in Applied Physics, Delft University of Technology, Delft, The Netherlands; 2 Department of Systems Biology, University of Massachusetts Chan Medical School, Worcester, MA USA

## Correction to: Journal of Biological Physics (2021) 47(4):355–369 https://doi.org/10.1007/s10867-021–09592-7

The article “Predictive landscapes hidden beneath biological cellular automata”, written by Lars Koopmans and Hyun Youk, was originally published Online First without Open Access. After publication in volume 47, issue 4, page 355–369 the author decided to opt for Open Choice and to make the article an Open Access publication. Therefore, the copyright of the article has been changed to © The Author(s) 2021 and the article is forthwith distributed under the terms of the Creative Commons Attribution 4.0 International License, which permits use, sharing, adaptation, distribution and reproduction in any medium or format, as long as you give appropriate credit to the original author(s) and the source, provide a link to the Creative Commons licence, and indicate if changes were made. The images or other third party material in this article are included in the article’s Creative Commons licence, unless indicated otherwise in a credit line to the material. If material is not included in the article’s Creative Commons licence and your intended use is not permitted by statutory regulation or exceeds the permitted use, you will need to obtain permission directly from the copyright holder. To view a copy of this licence, visit http://creativecommons.org/licenses/by/4.0/.

The original article has been corrected.

